# Comparative Prevalence Estimation and Phylogenetic Analysis of Novel Porcine Parvoviruses (PPV2–7) in Hungarian Pig Herds

**DOI:** 10.1155/2024/5117884

**Published:** 2024-11-15

**Authors:** Barbara Igriczi, Lilla Dénes, Kitti Schönhardt, Aleksandra Woźniak, Tomasz Stadejek, Gyula Balka

**Affiliations:** ^1^Department of Pathology, University of Veterinary Medicine, 1078, István Str. 2., Budapest, Hungary; ^2^National Laboratory of Infectious Animal Diseases, Antimicrobial Resistance, Veterinary Public Health and Food Chain Safety, University of Veterinary Medicine, 1078, István Str. 2., Budapest, Hungary; ^3^Department of Pathology and Veterinary Diagnostics, Institute of Veterinary Medicine, Warsaw University of Life Sciences—SGGW, Nowoursynowska 159C 02-776, Warsaw, Poland

**Keywords:** genetic variability, oral fluid, porcine parvoviruses, processing fluid, viral prevalence

## Abstract

To date, seven novel parvoviruses have been identified in pigs and designated as porcine parvovirus 2–7 (PPV2–7). The presence of these emerging viruses has been reported in several countries around the world, although their pathogenic role and clinical and economical relevance are largely unknown. Here, we report the estimated prevalence and genetic diversity of novel PPV2–7 in Hungarian pig herds and the detection of these viruses in two Slovakian pig farms. For the comparative prevalence estimation, 2505 serum samples from different age groups, 218 oral fluid samples, and 111 processing fluid samples were collected from 26 large-scale Hungarian farms according to a systematic, cross-sectional sampling protocol. All samples were tested by real-time quantitative polymerase chain reaction (qPCR), and the presence of at least one PPV was detected in 24 of the 26 (92%) Hungarian and both Slovakian farms, suggesting high levels of subclinical circulation in most herds. The estimated PPV2–7 prevalence in Hungary varied from 50% to 89%, with PPV4 being the least and PPV2 being the most prevalent virus. The highest detection rates were observed in oral fluid samples, indicating that this sample type is most suitable for screening PPVs, but all viruses were also detected in serum samples and processing fluids. All novel PPVs were most frequently detected in the serum samples of weaned pigs and fatteners, with slightly higher viral burden in the younger age groups. These results may suggest an age-related susceptibility, which could play a significant role in the epidemiology of these viruses, impacting herd health and productivity.

## 1. Introduction

Porcine parvoviruses (PPVs) are important topics of veterinary virology due to their widespread prevalence and impact on the global swine industry. The first known PPV, the PPV1, identified in the 1960s, is a significant, well-researched pathogen causing reproductive failure in breeding herds [1]. However, recent advances in molecular diagnostics have led to the identification of several new PPVs, termed PPV2 through PPV8 [2–8].

PPVs are small, nonenveloped viruses with linear single-stranded DNA genomes ranging from 4 to 6.3 kb, which contain two major open reading frames (ORFs). ORF1 encodes non-structural (NS) proteins necessary for viral replication (NS), and ORF2 encodes viral capsid proteins, viral protein (VP) [9]. PPVs belong to the *Parvoviridae* family which is divided into three subfamilies based on their host specificity: the *Parvovirinae* and the *Hamaparvovirinae* infecting vertebrates and the *Densovirinae* infecting arthropods. Within *Parvovirinae*, there are three types of PPVs, classified into genera: *Protoparvovirus* (PPV1 and PPV8), *Tetraparvovirus* (PPV2 and PPV3), and *Copiparvovirus* (PPV4 and PPV6). The *Hamaparvovirinae* subfamily contains the *Chapparvovirus* genus, which includes PPV7. PPV5 remains unclassified, but due to its close relation with PPV4, it is tentatively assigned to the *Copiparvovirus* genus [10].

Subsequent discoveries of novel PPVs expanded the spectrum of PPVs, each exhibiting distinct epidemiological and clinical characteristics. Studies on these newly identified viruses indicate their widespread prevalence globally [11–22], but the pathological consequences of the infections are poorly understood. PPVs were often found circulating among healthy pigs [13–15, 17, 18], but in some cases, they were detected in animals displaying different clinical symptoms [16, 20, 23, 24]. Among the novel PPVs, only PPV2 is currently considered a potentially pathogenic virus. The frequent detection of PPV2 in the lung tissues of pigs suffering from respiratory disease suggests that it may play a role in the development of porcine respiratory disease complex (PRDC) in the infected animals [24, 25]. In addition to PPV2, most PPVs are frequently identified in complex diseases like porcine circovirus-associated disease (PCVAD) or PRDC as coinfections with other bacterial and viral pathogens, such as porcine circovirus 2 (PCV2) or porcine reproductive and respiratory syndrome virus (PRRSv) [16, 20]. These novel PPVs may facilitate the replication of primary pathogens in infected animals and act as cofactors for the occurrence and severity of various multifactorial diseases.

PPV2, first identified in 2001 in serum samples used for hepatitis E virus research in Myanmar, has been detected in various countries indicating its global distribution [2, 18, 26]. The high prevalence of PPV2 in pigs with respiratory diseases and its predominant association with macrophages in the lungs suggest that it likely plays a role in the development of PRDC [24]. Another novel PPV with a worldwide distribution is PPV3, which was originally identified as porcine hokovirus in Hong Kong in 2008 alongside other bovine parvoviruses [3]. PPV4 and PPV5 were reported in 2010 and 2013, respectively, in the United States. PPV4 was identified in the lung lavage of a diseased pig coinfected with PCV2, while PPV5 was first detected in lung samples during a PPV4 prevalence screening [4, 26]. PPV6 was initially identified from aborted pig fetuses in China in 2014 [6]. PPV4, PPV5, and PPV6 seem to be closely related, and they do not cluster with the three previously identified PPVs [10]. PPV7 was first identified in 2016 by metagenomic sequencing of rectal swabs from healthy pigs without clinical symptoms [7], and PPV8 was detected in 2022 by high-throughput sequencing (HTS) of PRRSv-positive samples [8].

Among these novel PPVs, in the Hungarian pig population so far, the presence of PPV2, PPV3, and PPV4 has been reported [13, 27]. In the present study, our aim was to screen large-scale pig herds in a systematic way to assess the prevalence of all novel PPVs (PPV2–7) and to investigate the genetic diversity of the Hungarian PPV strains. While PPV8 was identified after the completion of these experiments [28], the focus of this study remains on PPV2–7. Comparing the presence and quantity of the viral DNA in different age groups and different diagnostic matrices provides a detailed insight into the circulation of the novel PPVs within the farms, which might help better understand the biology and epidemiology of these viruses.

## 2. Materials and Methods

### 2.1. Sample Collection

In this research, samples were gathered from 26 large-scale pig farms in Hungary between 2020 and 2023, as a part of an active surveillance sampling (ethical permission number: PE/EA/544-5/2018) [28–30]. Additionally, we acquired samples from two Slovakian pig farms near the Hungarian border for diagnostic purposes. The farms were all farrow-to-finish varied in terms of sow herd size (from 520 to 2200), genetics, and basic production parameters. The participation in the sampling campaign was voluntary, regardless of their health status, and no significant clinical disease was reported in the herds during the sampling period. On each farm, 10 blood samples were drawn from 2–, 4–, 6–, 8–, 10–, 14–, and 18-week-old pigs, gilts, and sows of two and four parities. Five pen-based oral fluid samples were collected from weaned pigs (8–12 weeks of age (WOA)) and from fatteners (18–20 WOA). For oral fluid sample collection, cotton ropes were placed in each pen for the pigs to chew on. After 15–20 min, the ropes were collected, and the liquid from each piece was extracted into plastic tubes. Five processing fluid samples were taken from each sow farm. Processing fluid samples were collected during piglet castration, with each bag of sample containing testicles and fluids from about 10 L. The number of serums, oral fluid, and processing fluid samples varied in some instances (Table S1). Altogether, 2505 serum samples, 218 oral fluid, and 111 processing fluid samples were collected, and all were stored at –80°C until further use.

### 2.2. Sample Processing and DNA Extraction

Prior to DNA extraction, the processing fluid and oral fluid samples were centrifuged, and equal volumes (100 µL) of serum samples were pooled by 5, so on each farm, two pools represented each age group. The DNA was extracted from 200 µL of oral fluid, processing fluid, or pooled serum samples by QIAcube automatic nucleic acid extractor (Qiagen, Hilden, Germany) using the QIAmp cador Pathogen Mini Kit according to the manufacturer's protocol. Nucleic acids were stored at –80°C until further analysis.

### 2.3. Real-Time Polymerase Chain Reaction (PCR) Detection of PPV2–7

Real-time quantitative PCR (qPCR) was used to detect viral DNA in the samples. The PCR assays were run on Q qPCR machine (Quantabio, Beverly, MA, USA). For PPV2–7 detection duplex, qPCR methods were used, in the combinations of PPV2 + PPV7, PPV3 + PPV6, and PPV4 + PPV5 [18]. The reaction mixture in all three cases contained PerfeCTa MultiPlex qPCR SuperMix (Quantabio, Beverly, MA, USA), extracted DNA, specific primer pairs for the two different viruses (300 nM), and two different probes (6-carboxyfluorescein [FAM]–hexachloro-fluorescein [HEX]-labeled probes in pairs, in 250 nM concentration). All primers and probes are listed in Table S2. The following temperature profile was used in all duplex qPCR reactions: 50°C for 2 min, then 95°C for 10 min, followed by 40 cycles of 95°C for 15 s, and then 60°C for 60 s. Samples with cycle threshold (Ct) values higher than 37 were considered negative.

The statistical analysis of the qPCR results was performed using GraphPad Prism 8. The comparison of the PPV2–7 prevalence in the different diagnostic matrices and age groups was performed by Fisher's exact test. The Ct values of different age groups and sample types were investigated using Mann–Whitney test by pairwise comparison. Statistical significance was set at *p*  < 0.05.

### 2.4. PPV2–7 Sequencing and Phylogenetic Analysis

Serum samples from PPV2–7-positive pools were extracted and tested individually. Sequencing reactions were attempted on the selected samples with low (<30) Ct values. The full capsid gene for PPV2, PPV3, and PPV4 and the full NS1 gene for PPV5, PPV6, and PPV7 were amplified as two, three, or four overlapping amplicons using two, three, or four sets of primers, respectively (Table S3). In each reaction, 5 µL extracted DNA was added to a mix of 5× Phusion HF Buffer (Thermo Scientific), 200 µM dNTPs, 1 µM of each primer, and 0.5 units of Phusion High-Fidelity DNA Polymerase. The reactions were run in a Genesy 96T gradient PCR machine (Tianlong, China), and the PCR products were visualized by agarose gel electrophoresis. The amplicons with the correct length were manually cut and purified using QIAquick Gel Extraction Kit (Qiagen, Hilden, Germany) according to the manufacturer's protocols. Capillary electrophoresis was performed by a commercial provider (BIOMI Kft., Gödöllő, Hungary).

Visualization and trimming of all chromatograms was performed using the Chromas 2.6.6 software (Technelysium Pty Ltd, South Brisbane, Australia). Both forward and reverse sequences were assembled and aligned using the E-INS-I method of the MAFFT version 7 online software [31]. A comprehensive alignment was conducted against the respective PPV2–7 reference sequences of various origins obtained from GenBank. For phylogenetic analysis, maximum likelihood trees were constructed using MEGAX software [32], performing 1000 replicates of bootstrap analysis. The PPV2–7 sequences identified in this study have been deposited in the National Center for Biotechnology Information (NCBI) GenBank under the accession numbers PP729185–PP729211.

## 3. Results

### 3.1. Geographical Distribution and Detection Rates of Novel PPVs in Hungarian Herds

**Figure 1:**
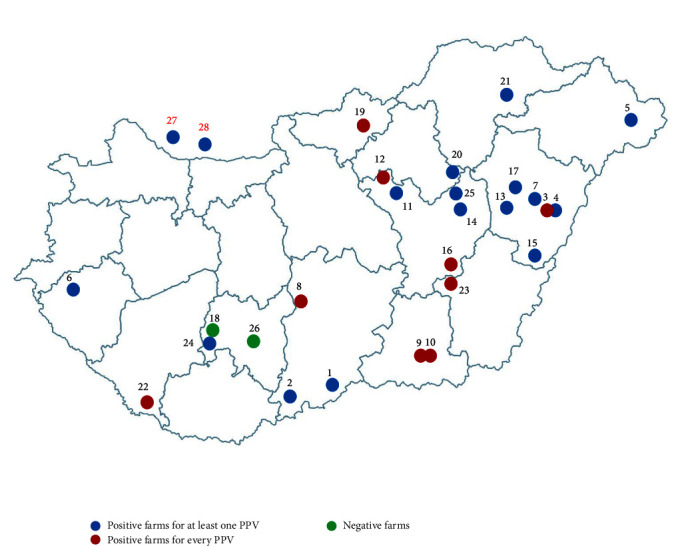
Map of Hungary showing the geographic location of the sampled pig farms included in our study. Farms are marked with different colors to indicate their infection status: green represents farms that tested negative for all porcine parvoviruses (PPVs), and blue and red indicate farms positive for only a few (1–6) or all PPVs, respectively. The Hungarian herds are labeled with black numbers, while the two Slovakian herds are labeled with red numbers.

At least one novel PPV was detected in 24 of the 26 (92%) sampled Hungarian pig farms included in this study (Figure 1). All novel PPVs were detected in at least one sample type in nine farms. In the 24 positive farms, circulation of one to five PPVs was detected, with different prevalence rates. One farm was positive only for PPV2, three farms were positive for three PPVs, three farms were positive for four PPVs, and on eight farms, the coexistence of five different PPVs were detected. When comparing viruses, PPV2 was detected in 23 farms (89%), PPV3 in 19 (73%), PPV4 in 13 (50%), PPV5 in 18 (69%), PPV6 in 21 (81%), and PPV7 in 22 (85%) farms, but the prevalence rates within each herd significantly varied: the exact number of PPV2–7 positive samples for each farm is detailed in Table S1. The two Slovakian farms, close to the Hungarian border, were also positive for most PPVs, as on Farm 27 we detected every PPV in at least one type of sample, except for PPV5, and on Farm 28, we also detected all PPVs except for PPV4. Interestingly, both PPV2- and PPV7-negative herds were located in one county, relatively close to each other, but there were no significant differences in the geographical distribution of the viruses.

### 3.2. PPV2–7 Detection in Different Sample Types

**Figure 2:**
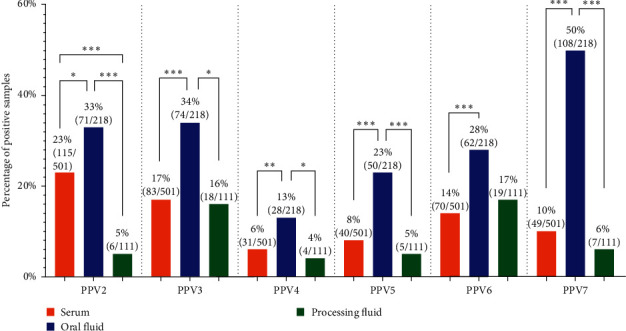
Percentages of porcine parvovirus 2–7 (PPV2–7)-positive serum pools (*n* = 501), oral fluid (*n* = 218), and processing fluid (*n* = 111) samples. The asterisks above the columns represent the statistically significant differences between diagnostic materials (⁣^*∗*^*p*  < 0.05, ⁣^*∗∗*^*p*  < 0.01, and ⁣^*∗∗∗*^*p*  < 0.001). The statistical comparison was performed using the Fisher's exact test.

Figure 2 shows the different prevalence rates of PPVs observed across the three diagnostic matrices examined in our study. The most frequently detected virus in serum samples was PPV2, with almost one-quarter (23%) of the samples testing positive. As for the other PPVs, altogether, 17% of the serum pools was positive PPV3, 6% for PPV4, 8% for PPV5, 14% for PPV6, and 10% for PPV7. The detection rates in the serum sample pools on farm level varied from 3% to 70%, which means that these viruses are present in almost all examined herds although their within-herd prevalences vary considerably.

Regarding the oral fluid samples, the positivity rates were relatively high: 33% for PPV2, 34% for PPV3, 13% for PPV4, 23% for PPV5, 28% for PPV6, and 50% for PPV7, with farm-specific detection varying from 8% to 100%. In the case of the processing fluid samples, the positivity percentages were 5% for PPV2, 16% for PPV3, 4% for PPV4, 5% for PPV5, 17% for PPV6, and 6% for PPV7, with a detection range on each farm from 10% to 100%.

Comparing the different diagnostic materials, the prevalence of all PPVs was significantly lower in serum samples than in oral fluids. No significant differences were observed comparing the serum and processing fluid samples, except for PPV2 where the detection rates in serum samples were significantly higher. The prevalence of all PPVs in oral fluids was also significantly higher than in processing fluids, except for PPV4 and PPV6 where no significant differences were observed.

**Figure 3:**
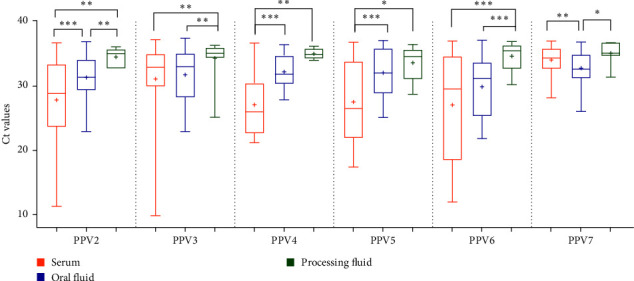
Distribution of the Ct values from porcine parvovirus 2–7 (PPV2*–*7)-positive serum (*n* = 501), oral fluid (*n* = 218), and processing fluid (*n* = 111) samples. The whiskers of the boxplots show the minimum and the maximum, and the average values are indicated with “+” signs. The boxes represent the interquartile range, with horizontal lines indicating the median values. The asterisks above the columns represent the statistically significant differences between diagnostic materials (⁣^*∗*^*p*  < 0.05, ⁣^*∗∗*^*p*  < 0.01, and ⁣^*∗∗∗*^*p*  < 0.001). The statistical comparison was performed using the Mann–Whitney test.

As shown in Figure 3, the comparative analysis of Ct values across the different diagnostic materials revealed distinct patterns. Notably, serum samples generally exhibited the lowest Ct values, while processing fluids consistently showed the highest. In most cases, statistically significant differences were found between the three sample types, indicating that the choice of the diagnostic material can significantly influence detection sensitivity. The mean Ct values in serum pools were ranging from 27.06 ± 8.05 (PPV6) to 34.06 ± 2.24 (PPV7) with the values representing the mean and standard deviation (SD). In contrast, oral fluid samples demonstrated slightly higher mean Ct values: from 29.88 ± 4.67 (PPV6) to 32.78 ± 2.51 (PPV7). The highest mean Ct values were observed in processing fluids, 33.61 ± 2.95 (PPV5) to 35.73 ± 0.94 (PPV7).

PPV2, PPV4, PPV5, and PPV6 were detected with significantly lower Ct values compared to PPV3 and PPV7. These results suggest that the high levels of viremia observed in individual animals can substantially lower the Ct values of serum pools. When comparing the distribution of Ct values measured in oral fluids and processing fluids, no significant differences were found between the different viruses, except between PPV6- and PPV7-positive oral fluid samples. These two diagnostic matrices represent a larger number of animals per sample and are more prone to contamination, which likely accounts for the higher Ct values observed. Despite these differences, all sample types proved to be effective for PPV screening.

### 3.3. PPV2–7 Detection Rates in Different Age Groups

**Figure 4:**
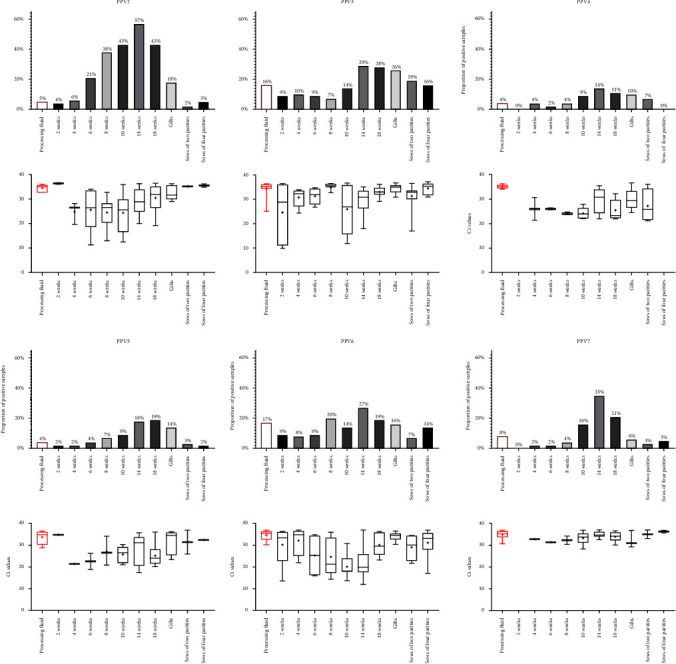
The upper diagrams show the percentages of porcine parvovirus 2–7 (PPV2*–*7)-positive processing fluid and serum samples of different age groups. The lower diagrams display boxplots of the Ct values from PPV2–7-positive processing fluid and serum samples, showing the distribution of viral loads in different age groups. The whiskers of the boxplots show the minimum and the maximum, and the average values are indicated with “+” signs. The boxes represent the interquartile range, with horizontal lines indicating the median values.

Almost all PPVs were found in every tested age group with detection rates varying between 2% and 57%. PPV4 was absent from the serum samples belonging to the sows of four parities, and both PPV4 and PPV7 were not detected in the 2-week-old age group, but other than that, there were at least one positive sample in each age group for every novel PPV. The percentages of the positive samples in each age group and the corresponding Ct values are shown in Figure 4. The highest detection rates were observed in the samples of the fatteners (14 and 18 WOA) but also the weaned pigs (6, 8, and 10 WOA). The findings in serum samples were consistent with those in oral fluids, as the oral fluid samples were collected from age groups (8–12 WOA and 18–20 WOA) that also showed high positivity rate in serum samples. The higher positivity rates detected in oral fluid samples may be attributed to the collective nature of the sample, as it represents a larger population of animals simultaneously. No significant differences were observed between the two age groups in terms of detection frequencies or viral loads in oral fluids.

PPV2 was the most prevalent of all PPVs, with exceptionally high positivity rate in the 10- (43%), 14- (57%), and 18-week-old (43%) age groups. PPV3 and PPV6 were also common in the tested farms; almost one-third of the fattener's samples were positive for each virus. Interestingly, PPV3 and PPV6 were the most common in processing fluid samples (16% and 17%, respectively), which represent the youngest, newborn age group in our study. All three PPVs mentioned above were detected with relatively low Ct values, especially in the younger age groups such as the 2- and 4-week-old piglets and weaned pigs, which suggests high levels of viremia in some of the pigs included in these pools. PPV4, PPV5, and PPV7 were the least frequently detected PPVs, and the Ct values of the positive samples were also higher compared to the other three PPV types. In a few farms, where a relatively large proportion of serum samples tested positive for one of these viruses, we were able to detect low Ct values—high viral copy numbers even for these PPVs.

### 3.4. Phylogenetic Analysis of Novel PPVs

**Figure 5:**
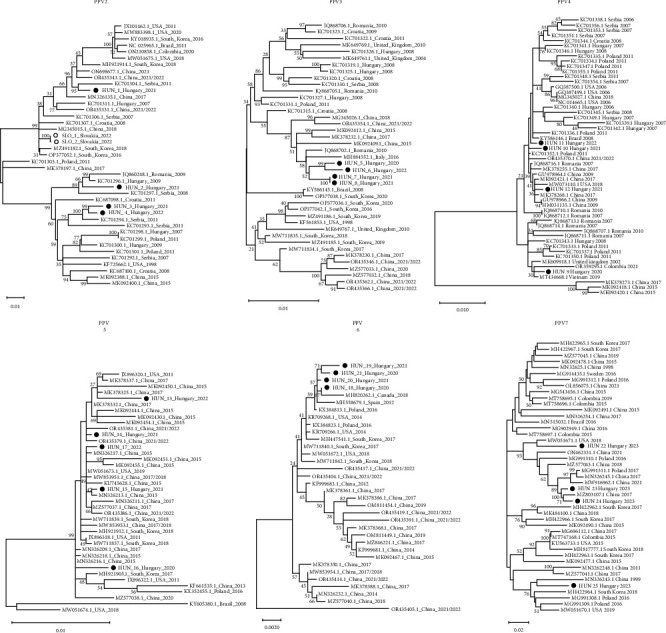
Phylogenetic analysis of the VP1 gene of porcine parvovirus (PPV)2, PPV3, and PPV4 and the NS1 gene of PPV5, PPV6, and PPV7. All Hungarian PPV sequences are marked with black dots, and the Slovakian PPV2 sequences are marked with black circles. The National Center for Biotechnology Information (NCBI) accession numbers corresponding to our sequences are summarized in Table S4. The phylogenetic analysis was performed using the MEGAX software, employing the maximum likelihood method, and it was supported by 1000 bootstrap replicates to ensure the robustness of the analysis. Reference sequences for VP1 and NS1 were acquired from GenBank and then aligned with the sequences identified in our study for comparison. NS, nonstructural; VP, viral protein.

Four nearly complete capsid sequences for PPV2 were determined in samples from various Hungarian pig farms. Additionally, two PPV2 sequences were identified from Slovakian samples. All PPV2 sequences from this study lack a small fragment (24–31 nucleotides) at the gene's end. However, the variable region of the VP1 capsid gene, crucial for accurate phylogenetic analysis, was fully sequenced in each case. The maximum likelihood phylogenetic tree revealed two main clades, incorporating 35 sequences from GenBank alongside our PPV2 sequences, with nucleotide identity ranging from 92.72% to 98.44% across clades (Figure 5). Three sequences from Hungary clustered in the second clade, comprising mainly European samples. Only one Hungarian and both Slovakian sequences grouped in the first clade, characterized by a more diverse geographical representation, including sequences from the United States, Brazil, South Korea, and China. Comparative nucleotide sequence analysis showed 93.49%–98.81% similarity among Hungarian capsid genes and 93.52%–96.89% between Hungarian and Slovakian strains.

For PPV3, we sequenced four complete capsid genes and aligned them with 37 PPV3 sequences of varied origins. The phylogenetic tree displays two clades with nucleotide identities between 97.88% and 99.46% (Figure 5). The Hungarian sequences clustered together in the first clade, which included mainly European and some Chinese strains, while the second clade was primarily composed of Chinese and South Korean strains. Comparative analysis revealed 99.24%–100% similarity among the capsid genes of the Hungarian strains.

Regarding PPV4, we determined the complete viral capsid gene sequences from four Hungarian samples, and the PPV4 phylogenetic tree showed two tentative clades, including 50 sequences from GenBank and our PPV4 sequences, with overall nucleotide identities ranging from 95.01% to 99.77% (Figure 5). All Hungarian sequences belong to the first clade with the majority of sequences, while the second clade only consists of three Chinese strains. Comparative sequence analysis indicated 99.03%–99.54% similarity among the Hungarian strains' capsid genes.

We also determined the complete NS1 gene sequences from PPV5, PPV6, and PPV7 positive Hungarian samples and performed phylogenetic analyses. The phylogenetic trees for PPV5 and PPV6 revealed two tentative clades, while for PPV7, four tentative clades were identified, incorporating 30–41 sequences downloaded from GenBank (Figure 5). The Hungarian PPV5 and PPV6 sequences showed high nucleotide identity (97.18%–99.89% and 97.75%–99.9%, respectively) across clades, reflecting a high degree of sequence similarity, with notable diversity in geographic origin for the sequences in the first clades. Comparative nucleotide sequence analysis indicated high similarity among the Hungarian strains. For PPV7, the Hungarian sequences fell into two different tentative clades indicating close genetic relations with sequences from Poland, China, and the United States, reflecting a broad genetic diversity among PPV7 strains. Comparative analysis showed 92.56%–98.34% overall nucleotide identities between all sequences and 93.36%–98.6% similarity among the Hungarian strains.

## 4. Discussion

Until the early years of the twenty-first century, PPV1 stood as the sole recognized member of PPVs, acclaimed for its extensive research history and established role as a major reproductive pathogen. Since then, seven novel PPVs have emerged in swine populations, which are proven to be widely distributed worldwide, though their potential pathogenic role remains a subject of ongoing investigations. In this study, our aim was to assess the prevalence and circulation patterns of novel PPVs in the Hungarian pig population and to perform phylogenetical analyses on the Hungarian PPV2–7 strains.

Our results showed that all novel PPVs are widespread in Hungary, as at least one of them was detected in 24 of the 26 (92%) Hungarian farms involved in our study (Figure 1). The estimated PPV2–7 prevalence in Hungary varied from 50% to 89%, with PPV4 being the least and PPV2 being the most prevalent virus. The comparison of the different diagnostic matrices used in our study showed that all PPVs were more frequently detected in oral fluids than in serum or processing fluids. These results align with the findings of a similar study conducted in Poland, where high detection rate of PPVs was observed in oral fluid samples during the examination of a large number of serum, oral fluid, and fecal samples [18]. However, the use of different sampling protocols, different sample types, and the varying age, health status, and number of the sampled animals significantly complicate the interpretation of our findings in comparison to prior research conducted on novel PPVs.

For serum samples, the detection rates of all novel PPVs were usually similar or slightly different than (around 5%–20% positivity) what was observed in different countries [13, 17, 18, 20, 33–35]. In their 2012 publication, Cságola et al. [13] examined the Hungarian prevalence of PPV2, PPV3, and PPV4 in different sample types including 111 serum samples. They observed a significantly lower prevalence, with only 5.4% of the serum samples testing positive for PPV2. For PPV3 and PPV4, the proportion of positive serum samples was only slightly lower than our results (14.4% and 2.7%, respectively). The higher prevalence rates detected in our study suggest that these viruses become more widespread in domestic herds over the last decade. However, it is important to note that the difference may also be attributed to the smaller sample size and the fact that they did not use pooled serum samples. In the cases of PPV5, PPV6, and PPV7, our results represent the first scientific evidence of the presence of viruses in Hungary.

When analyzing PPV-positive oral fluids, we found that except for PPV3, the prevalences were slightly lower than what was reported in a similar study conducted in Poland [18]. The consistently high detection rates in this diagnostic material confirm that oral fluids are a reliable tool for the surveillance of PPVs. To our knowledge, this is the first study where processing fluid samples were used to detect and estimate the prevalence of PPV2–7 by comparing them with other diagnostic matrices. Despite generally observing relatively low detection rates, it was possible to successfully identify all PPVs in processing fluids. Interestingly, PPV6 which might be linked to reproductive failures [6] showed the highest prevalence in processing fluid samples. Comparing the reproductive parameters of the PPV6-positive farms at the time of the samplings, we found that the farm with one of the highest within-herd PPV6 prevalence also had the highest mortality rates in the farrowing unit (16.2%) and another farm with similarly high prevalence had a lower-than-average conception (85%) and farrowing rate (70%). A recent study conducted on Northern Italian pig farms demonstrated that PPV6, along with other PPVs, is frequently detected and potentially associated with reproductive failures, emphasizing its prevalence and relevance to pig health [23].

Our findings indicate that screening oral and processing fluids provides a practical approach for confirming the presence of PPV2–7 within a specific herd and monitoring PPV2–7 infections in newborn piglets also. On the other hand, it must be taken into consideration that these sample types represent a large number of animals at once. According to our protocol, one oral fluid sample represents a whole pen, and one processing fluid sample represents approximately 50–90 newborn male piglets; therefore, the exact prevalence of these viruses on herd level cannot be precisely determined. From the novel PPVs, only PPV3 was reported in Slovakia, where the authors found 19.1% PPV3 positivity amongst the 194 wild boars tested [36]. On the two Slovakian farms involved in our study, all novel PPVs were detected, which represents the first scientific evidence of the presence of these viruses, except for PPV3, in the country. All PPVs were detected in serum samples, all PPVs except PPV4 were present in oral fluid samples, and four PPV types (PPV3, PPV4, PPV6, and PPV7) were detected in processing fluid samples from the Slovakian herds.

The sampling protocol in this study covered most age groups and sow parities within the examined herds, so we could efficiently monitor the presence of the novel PPVs across all age groups. By using a pen- and litter-representative oraland processing fluids and pooled serum samples, we were able to efficiently gather data on viral loads across multiple animals in a cost-effective manner. According to our results, all PPVs were detected in all age groups, with notable variations in virus prevalence among them, and slightly higher viral burdens were observed in younger animals in certain cases (Figure 4). As processing fluids represent the youngest age group, the lower detection rate in this diagnostic matrix as well as in the serum samples of the 2- and 4-week-old piglets may be due to maternal immunity and adequate amounts of passively acquired antibodies, or it might be that most of these viruses primarily are not transmitted through vertical transmission. Based on our results, vertical transmission can be primarily assumed for PPV3 and PPV6, as these viruses were found with high frequency in both processing fluid samples and the sows' serum. The protective role of maternally derived antibodies has only been proven for PPV2 [37], but it can be speculated that it plays an important role in PPV3–8 infections also. A gradual increase of the relative infection rates in 6-, 8- or 10-week-old animals suggests waning protection of the maternal immunity which has also been described for several other viruses [29, 38–41]. Moreover, weaning stress could increase susceptibility to infections [42–44]. The highest detection rates observed for all PPVs in 14- and 18-week-olds may relate to regrouping in fattening pens. These results align with other PPV2–7 prevalence studies, where the authors usually described lower prevalence rates in suckling and weaned pigs and higher in fatteners and finishing animals [5, 16, 17, 19, 37, 45, 46].

The phylogenetic analysis and classification of novel PPVs are hindered by the limited number of reported sequences from various geographical origins. As more diverse full genome sequences become available, more accurate classifications can be proposed. Our phylogenetical analyses based on the capsid genes of PPV2, PPV3, and PPV4 showed that all three viruses form two distinct clades when compared to a representative group of sequences (Figure 5). Similar studies of PPV2 to date have also identified two distinct clades alongside relatively high nucleotide divergence [13, 16, 21]. This high level of divergence can be attributed to the high mutation rates of PPVs, which is comparable to ssRNA viruses, but recombination events and selective pressure may also contribute to their rapid evolution [12, 47]. Overall, it can be noted that Chinese sequences for all three PPVs are distributed across both clades, whereas European sequences often cluster together forming separate groups either with or in close proximity to each other. It is possible that similar to PPV7 [48], the Chinese strains of other novel PPVs are the ancestral strains and have a longer evolutionary history and greater genetic diversity compared to European strains, which may explain their more scattered placement in the phylogenetic tree. Previous studies have shown that PPV2 exhibits significant gene flow and recombination across regions, while PPV3 and PPV4 show a diversification reflected by the accumulation of geographically structured polymorphisms, which may explain the closer clustering of European strains [12]. Our newly identified sequences show a clustering pattern similar to that observed in the first detection of Hungarian strains: however, our sequences diverge from those previously described in Hungary, bearing a closer resemblance to more recent sequences from Italy, Poland, Romania, and Serbia. This is presumably due to the rapid evolutionary rate of these viruses, since these countries are not major exporters of live piglets to Hungary [49]. Cságola et al. [13] reported no differences in Hungarian PPV4 capsid sequences. In contrast, our study identified several nucleotide differences among the sequences, although the similarity among them is more than 99%; PPV4 still appears to be the most conserved when comparing the capsid genes of PPV2–4. Further analysis of the NS1 gene of PPV5, PPV6, and PPV7 demonstrates that this region of the viral genome is quite conserved in PPV5 and PPV6, while less so in PPV7, which is supported by a Chinese study indicating that PPV7 has a faster evolutionary rate than other PPVs [48]. Similarly, Milek, Wozniak, and Stadejek [19] reported a similarly high level of diversity among PPV7 NS1 sequences detected in Poland. For PPV2 and PPV7, we observed that the two Slovakian and some Hungarian sequences show closer relationships to strains from China or America than to each other, indicating their affiliation with distinct genetic lineages and suggesting a prolonged evolutionary history in Europe. Conversely, for the other four viruses, our results indicate a slower evolutionary rate.

Our study's limitations include insufficient available information regarding the epidemiological connections between Hungarian farms and pig farms abroad. Despite detecting all these viruses in clinically healthy animals, we have to keep in mind that coinfections with different viruses can cause various problems, for example, interfering against an infecting virus, increasing the virulence or replication of a virus, and leading to recombination events [50]. Moreover, novel PPVs can potentially play important roles as viral cofactors in developing complex diseases such as PCVAD or PRDC [20, 51]. As some of these viruses have been also linked with reproductive disorders [6, 52] or respiratory diseases [24] in swine, it is important to determine the pathogenesis of these novel viruses. Our further research will investigate the correlation between different pathologies and the presence and viral burden of these novel PPVs in clinically ill animals.

## 5. Conclusion

This study establishes the broad distribution of novel PPVs PPV2–7 within the Hungarian pig population, revealing diverse prevalence across multiple diagnostic matrices. Notably, these viruses are consistently detected across all age groups, indicating subclinical circulation within the examined herds. Oral fluids, in particular, show higher detection rates, highlighting the significance of this noninvasive sample type in screening for these emerging pathogens. Phylogenetic analysis indicates high genetic diversity among some PPV strains, particularly for PPV2 and PPV7, reflecting the dynamic evolution of these viruses within the swine population.

## Data Availability

Sequence data gathered during the study can be accessed under GenBank accession numbers PP729185–PP729211. Data regarding the name and exact location of the farms involved in the study are confidential due to business secret of the owners.

## Supporting Information

Supporting Information Table S1: Summary of the examined Hungarian farms, sample types, and sample sizes. The table also includes the number and proportion of the PPV2–7-positive samples. Table S2: Specific primes and probes used for the qPCR detection of PPV2–7. Table S3: Specific primer sets used for sequencing the capsid or NS1 genes of PPV2–7. Table S4: The NCBI accession numbers and the corresponding Hungarian PPV2–7 strains as indicated on the phylogenetic trees.
